# A129 A RETROSPECTIVE ANALYSIS OF ENDOSCOPIC RETROGRADE CHOLANGIOPANCREATOGRAPHY IN PATIENTS WITH PANCREATICODUODENECTOMY

**DOI:** 10.1093/jcag/gwac036.129

**Published:** 2023-03-07

**Authors:** D Low, C Liu, F Shaukat Ali, A Al Nakshabandi, J Lee

**Affiliations:** MD Anderson Cancer Center, Houston, United States

## Abstract

**Background:**

Surgically altered anatomy remains a challenge when performing advanced endoscopic procedures including endoscopic retrograde cholangiopancreatography (ERCP). Post-surgical anatomy frequently encountered in clinical practice that affects ERCP success includes Billroth II, roux-en-y gastric bypass, roux-en-y hepaticojejunostomy and pancreaticoduodenectomy (PD). While ERCP has a success rate of 90-95% in patients with native anatomy, the data is less robust in patients with PD anatomy. Advancement of the endoscope to the surgical anastomoses remains challenging as PD anatomy requires accurate identification of the afferent limb, and encourages acute bowel angulation and loop formation. In addition, navigation to the anastomosis may be more challenging with a sideviewing endoscope. Although forward viewing endoscopes may more readily reach the surgical anastomosis, cannulation may be difficult without an elevator or device-appropriate instruments. As a result, a retrospective analysis of technical and clinical success rates in ERCP was analyzed in post-PD patients.

**Purpose:**

The purpose of this study was to evaluate the technical and clinical success of ERCP in patients with post-PD anatomy.

**Method:**

A retrospective analysis was conducted on all patients with post-PD anatomy referred to our institution between 2006 to 2021 for ERCP. Demographic and procedural details including gender, age, indications, primary malignancy, and anastomosis identification were collected. Major outcomes included technical success (TS) rate (successful biliary or pancreatic intervention) and clinical success (CS) rate (improvement in patient symptomatology or normalization of bilirubin level).

**Result(s):**

A total of 47 patients underwent 102 ERCPs for biliary (n = 98; 96.1%) and pancreatic (n = 4; 3.9%) indications. There were 31 (66.0%) male patients and 16 (34.0%) female patients. The average age of patients was 61.5 years. The most common primary malignancies included pancreatic adenocarcinoma (n = 26; 55.3%), pancreatic neuroendocrine tumour (n = 5; 10.6%), and ampullary adenocarcinoma (n = 4; 8.5%). The most common indications for ERCP included obstructive jaundice (n = 22; 21.6%), stent evaluation (n = 30; 29.4%), and cholangitis (n = 35; 34.3%). Surgical anastomosis was identified in 90 (88.2%) patients. The overall TS and CS rates were 82.4% and 75.5%, respectively. When adjusted for anastomosis identification, the overall TS and CS rates were 93.3% and 82.2%.

**Conclusion(s):**

Despite the challenges of conducting ERCP in patients with post-PD anatomy, this retrospective analysis demonstrates an adequate overall TS and CS rate. Although endoscope advancement to the surgical anastomoses may be challenging, TS and CS rates improved if the anastomosis was reached and identified. The advent of endoscopic enteroenterostomy formation, balloon-assisted enterscopy, and rigidizing overtubes may assist in reaching surgical anastomoses. As a result, consideration should be undertaken to attempt ERCP in post-PD patients.

**Please acknowledge all funding agencies by checking the applicable boxes below:**

None

**Disclosure of Interest:**

None Declared

